# Synthesis, characterization and stress-testing of a robust quillaja saponin stabilized oil-in-water phytocannabinoid nanoemulsion

**DOI:** 10.1186/s42238-021-00094-w

**Published:** 2021-09-23

**Authors:** Abhinandan Banerjee, Justin Binder, Rayan Salama, John F. Trant

**Affiliations:** 1 Department of Chemistry and Biochemistry, University of Windsor, 401 Sunset Ave., Windsor, ON, Canada; 2 Peak Processing Solutions, Tecumseh, ON, Canada

**Keywords:** Cannabis, Nanoemulsion, High pressure homogenization, Stress testing

## Abstract

**Background:**

This study describes the design, optimization, and stress-testing of a novel phytocannabinoid nanoemulsion generated using high-pressure homogenization. $\text {QNaturale}^{\circledR }$, a plant-derived commercial emulsifier containing quillaja saponin, was used to stabilize the lipid phase droplets in water. Stress-testing was performed on this nanoemulsion in order to evaluate its chemical and colloidal stability under the influence of different environmental factors, encompassing both physical and chemical stressors.

**Methods:**

Extensive optimization studies were conducted to arrive at an ideal nanoemulsion formulation. A coarse emulsion containing 16.6 wt% CBD-enriched cannabis distillate and 83.4 wt% carrier (soybean) oil dispersed in 10 wt% $\text {QNaturale}^{\circledR }$ (1.5 wt% quillaja saponin) solution after 10 homogenization cycles at a pressure of 30,000 psi produced a stable nanoemulsion. This nanoemulsion was then subjected to the stress studies.

**Results:**

The optimized nanoemulsion had an average droplet diameter of *ca.* 120 nm and average droplet surface *ζ* potentials of *ca.* -30 mV. It was imaged and characterized by a variety of protocols. It proved to be stable to droplet agglomeration and phase separation upon storage under ambient conditions for 6 weeks, as well as under a variety of physical stressors such as heat, cold, dilution, and carbonation. pH values ≤2 and moderately high salt concentrations (> 100 mM), however, destabilized the nanoemulsion, eventually leading to phase separation. Cannabis potency, determined by HPLC, was detrimentally affected by any changes in the nanoemulsion phase stability.

**Conclusions:**

Quillaja saponin stabilized cannabidiol(CBD)-enriched nanoemulsions are stable, robust systems even at low emulsifier concentrations, and are therefore significant from both a scientific as well as a commercial perspective.

**Supplementary Information:**

The online version contains supplementary material available at (10.1186/s42238-021-00094-w).

## Background

As the therapeutic benefits of cannabis become scientifically established, and the use of nutraceutical and recreational cannabis products is decriminalized in countries such as Argentina, Belgium, Georgia, Uruguay, South Africa, etc., cannabis research is expected to flourish in the new decade (McIver 2017). Canada, especially, is expected to play a pioneering role in cannabis research, given that it is the only G7 country to have completely legalized medical as well as recreational cannabis products, whilst simultaneously creating a robust regulatory system for the purposes of quality assurance and safe dissemination of cannabis-containing consumer products (Government of Canada 2018).

Cannabis is a highly complex mixture, consisting of over 120 phytocannabinoids with related chemical structures, along with a variety of terpenes and flavonoids (Turner et al. 2017). As many of these natural products interact with the central nervous system (CNS) receptors (Zou and Kumar 2018), cannabis could prove to be an exceedingly promising source for pharmacophore discovery. Amongst the phytocannabinoids, *Δ*^9^- tetrahydrocannabinol (THC) and cannabidiol (CBD) are by far the most abundant, and consequently, the most thoroughly examined (Hanus et al. 2016; Reekie et al. 2017). THC and CBD have demonstrated neuroprotective, immunomodulatory, as well as anti-inflammatory effects (Cameron and Hemingway 2020), leading to their inclusion as adjunctive treatment for malignant brain tumors, Parkinson’s disease, Alzheimer’s disease, multiple sclerosis, neuropathic pain, and childhood seizure disorders (Maroon and Bost 2018). Experimental studies are being conducted in order to examine anecdotal and preliminary scientific evidence of their benefits in alleviating psychiatric and mood disorders, such as schizophrenia, anxiety, depression, addiction, and post-traumatic stress disorder (Shahbazi et al. 2020; Scherma et al. 2018). While the psychoactivity of THC can be less desirable in some clinical settings, the non-psychoactive CBD, with its anti-inflammatory, anti-convulsive, and anti-emetic effects, is a prime candidate for the development of functional cannabinoid-based nutraceuticals (Parker et al. 2002; Khan et al. 2020). Other promising biological effects of cannabinoids include anti-obesity and antidiabetic effects of tetrahydrocannabivarin (THCV) (Abioye et al. 2020) and in developing fluoride- and alcohol-free anti-bacterial mouthwash and oral care products (Vasudevan and Stahl 2020).

CBD is highly lipophilic and hydrophobic, thereby making oral delivery routes for CBD an essential but underexplored field of research (Grotenhermen 2003). CBD has low oral bioavailability (*ca.* 6–9%) owing to its low aqueous solubility and its susceptibility to ready clearance during first pass metabolism; therefore, it is up to the formulation science community to devise new routes for orally administering cannabinoids to both increase and ensure reproducibility of blood plasma concentrations (Elgart et al. 2012). Protocols such as self-emulsifying drug delivery systems have been applied in the past to increase solubility and reduce first pass metabolism of lipophilic bioactives such as cannabinoids in the context of pharmaceutical preparations (Cherniakov et al. 2017).

A potential approach used to augment the bioaccessibility of orally administered lipophilic nutraceuticals is their incorporation into nanoemulsions (Jaiswal et al. 2015; Solans et al. 2005). Due to reduced droplet sizes and lower emulsifier concentrations in these systems relative to microemulsions or liposomes, loading of phytocannabinoids in the lipid phase of nanoemulsions may improve drug bioaccessibility (Acosta 2009), prevent oxidative degradation of the cannabinoids (Sun et al. 2015), and provide a more palatable and “label friendly” cannabinoid ingestible (Raikos and Ranawana 2017).

**Fig. 1:**
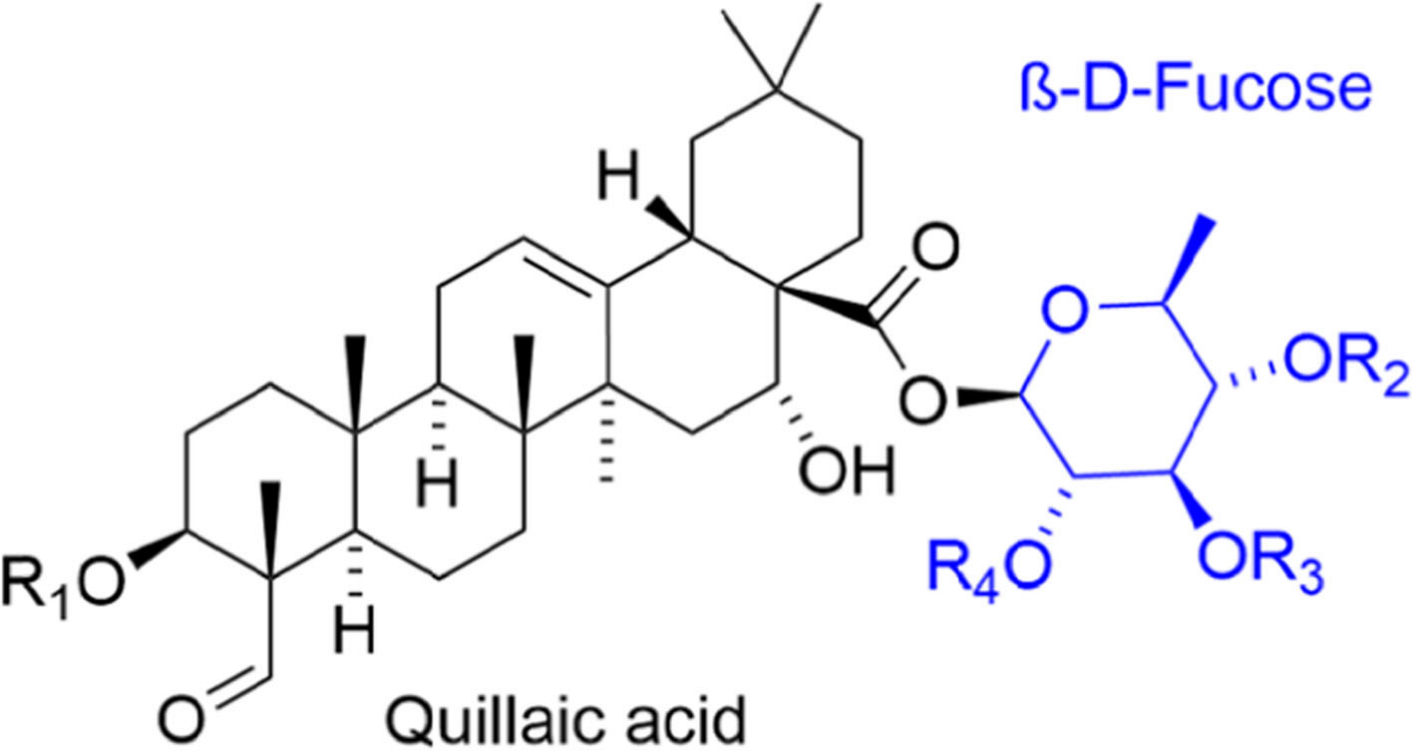
General chemical structure of Quillaja saponin; R _1,2,3,4_ represent saccharide or acyl residues. Adapted from Reichert et al. (2019)

A stable nanoemulsion has a bulk phase (here, water); a diffused phase (here, a solution of cannabis extract in an edible oil); and an emulsifier. The emulsifier is of paramount importance: not only does it prevent the lipid phase droplets from agglomerating and undergoing phase-separation, but it also dictates the taste and ‘mouthfeel’ of the resultant nanoemulsion (Mouritsen and Styrbaek 2017; Panghal et al. 2019). Excess emulsifier may very well imbue an emulsion with a bitter ‘chemical’ or metallic taste, or an unpleasant texture; moreover, cost, safety and regulatory issues also come into play. Naturally occurring, ‘label-friendly’ emulsifiers are therefore preferred when devising such nanoformulations. $\text {QNaturale}^{\circledR }$ is a commercially available emulsifier containing quillaja saponin obtained from the bark of the *quillaja saponaria* Molina tree. It has previously been found to be effective for the fabrication of nanoemulsions by high-energy methods such as microfluidization (Bai et al. 2016). Structurally, quillaja saponin might best be described as a mixture of triterpenoid saponins comprising a hydrophobic quillaic acid backbone glycosylated with hydrophilic sugar moieties (Fig. 1). The interfacially active saponins in quillaja extracts consist of two hydrophilic sugar chains and one hydrophobic aglycone, generating a three-unit structure with distinct hydrophilic–hydrophobic–hydrophilic properties (Reichert et al. 2019). The emulsion-stabilizing properties of $\text {QNaturale}^{\circledR }$ are attributed to strong electrostatic repulsion in quillaja saponin coated lipid droplets with high negative surface *ζ*-potentials between pH 3 and 9 (Kharat and McClements 2019; Zhu et al. 2019). Additionally, the fast adsorption kinetics of $\text {QNaturale}^{\circledR }$ onto lipid droplet surfaces also promotes the formation of stable quillaja saponin oil-in-water nanoemulsions with sub- *μ*m droplet sizes (Bai et al. 2016; Zhang et al. 2016). Another important component is the carrier oil, which modulates emulsion stability and bioactive bioaccessibility. For the purposes of this study, soybean oil, a biodegradable, non-toxic, and sustainably sourced edible oil was selected owing to its excellent miscibility with cannabis extracts (Hsu and Nacu 2003). Soybean oil and soy protein isolates have been used in the past as carrier media for pharmaceuticals (Takino et al. 1994; Chung et al. 2001) and nutraceuticals (Teng et al. 2012; Chen and Subirade 2009).

This report adds to the few published accounts of cannabinoid emulsions as potential medicinal and/or nutraceutical products. Recently, Marangoni et al. reviewed THC encapsulation and stability in food matrices (Marangoni and Marangoni 2019); similarly, McClements has reviewed scientific developments in the emerging field of cannabis-enriched foods (McClements 2020). In an in vivo study on bile-fistulated rats, Sato and colleagues examined a medicinal CBD emulsion containing very high doses of polyethylene glycol sorbitan monolaurate (tween-20) (70% w/w); the intestinal absorption of CBD and its pharmacokinetic profiles in rats were determined (Nakano et al. 2019). The effect of bile secretion on the intestinal absorption of CBD from oil and emulsion was also studied — these emulsions, however, were clearly unsuitable candidates for nutraceuticals owing to the excessive tween-20 content. Self-emulsifying drug delivery systems were utilized by Sacks and colleagues in 2018 for a novel THC/CBD capsule formulation (Atsmon et al. 2018). Similarly, Knaub et al. in 2019 studied a novel CBD nanoemulsion generated *in situ* within the human gastrointestinal tract; this formulation was based on VESIsorb® formulation technology (Knaub et al. 2019). However, the focus of these two studies were on oral bioavailability of cannabinoids in human test subjects rather than on the preparation of the CBD nanoemulsion. In 2020, Peshkovsky and colleagues published an account of a tween-80/span-80 stabilized CBD-containing nanoemulsions generated using a bench-scale ultrasonic liquid processor; they also demonstrated the scalability of their emulsification process (Leibtag and Peshkovsky 2020). Unfortunately, none of these studies employed a rational design approach suitable for the creation of nutraceutical-standard cannabis-enriched nanoemulsions; furthermore, the nanoemulsions were not assessed for colloidal and/or chemical stability and cannabis potency under non-ideal environmental conditions. The present study addresses this issue by rational design of a stable, robust CBD-infused nanoemulsion. Concomitantly, it examines the impact of parameters such as lipid-to-emulsifier ratio, composition of the lipid phase, and the number of homogenization cycles on the droplet sizes of the nanoemulsion and its CBD concentration values, *de facto* measures of the emulsion quality and stability. The resultant optimized nanoemulsion was then submitted to a variety of stressors mimicking production and storage conditions relevant to the beverage industry. Optical microscopy and scanning electron microscopy (SEM) are used to examine the impact of pH and additional electrolyte concentration on nanoemulsion droplet sizes, and by proxy, the overall stability of the system. This multi-factorial approach analyzing potential commercially-relevant cannabis nanoemulsions aims to provide us with detailed insight into their emulsion properties. This account of a stable, robust and plant-sourced cannabinoid nanoemulsion is expected is to be the first of a series of reports on the design, fabrication, and testing of cannabis-based nutraceutical products.

## Materials and methods

### Materials

$\text {QNaturale}^{\circledR }$ 200V (14–16 wt% quillaja saponin; Ingredion, New Jersey) was used as received. Soybean oil was sourced from Jeen International Corporation (New Jersey) and used without further purification. CBD-enriched cannabis distillate (CD_*CBD*_) was donated by XTRX (Ontario, Canada) and was used as received; the cannabinoid composition of the CD_*CBD*_ may be found in Table S1 (SI). The preservative potassium sorbate (Nantong Acetic Acid Chemical Co., China) was added to the coarse emulsions prior to high pressure homogenization. HPLC grade water (EMD Millipore) was used in all experiments. Sucrose and CaCl_2_ were purchased from Sigma Aldrich. For the measurement of pH, a freshly calibrated pH-meter (Milwaukee MW102 PRO+) was used. For the HPLC analysis, 1 mg/mL stock solution of each of the following cannabinoids was obtained from Cerilliant: cannabidiolic acid (CBD-A), tetrahydrocannabinolic acid (THC-A), cannabidiol (CBD), cannabigerolic acid (CBG-A), cannabigerol (CBG), cannabichromene (CBC), cannabinol (CBN), and delta-9 tetrahydrocannabinol (THC).

### Preparation of the nanoemulsions

A representative nanoemulsion was synthesized as follows: the CD_*CBD*_ (69% CBD; see Table S1) was magnetically stirred and heated in a water bath at *ca.* 65^∘^C, and to it was added the requisite weight of the soybean oil in order to create a homogeneous lipid phase. The stirring was continued for another 10–15 min. Meanwhile, $\text {QNaturale}^{\circledR }$ was dissolved in water in order to generate the aqueous phase. Finally, the lipid phase was added to the water phase in the correct proportion (usually, 10 wt% lipid phase in water) while subjecting the mixture to three cycles of blending using a high shear mixer (IKA-T-1000) at ambient temperatures (60 s on, 60 s off at power setting = 3). This generated a milky coarse emulsion, to which was added 0.1 wt% potassium sorbate, and enough citric acid to lower the pH to ∼3.4–3.8. The nanoemulsions were then formed by passing the coarse emulsions through a high pressure homogenizer (Nano DeBee, BEE International, USA; equipped with a chill loop) at a pressure of 30000 psi. The number of passes were optimized during the course of the study (*vide infra*). Finally, the nanoemulsions were poured in glass vials, capped, and stored either in a fridge at ∼4^∘^C in amber vials (for long term storage) or under ambient conditions in the dark (for short-term storage). The CBD concentration of the final emulsion was determined to be 10–11 *mg*.*g*^−1^ (slight variations are attributed to individual lots of emulsions made) on the basis of measured weights, although quantitative analysis of the nanoemulsion revealed slight loss of CBD during the emulsion preparation (*vide infra*).

### Emulsion droplet size distributions

In order to determine lipid particle size distributions in the nanoemulsions, dynamic light scattering (DLS), also known as photon correlation spectroscopy, was applied. This technique generated Z-average diameters of the dispersed phase droplets (*d*_*z*_), as well as the polydispersity index (PDI). Diluted samples (50- to 100-fold dilutions) were used to avoid multiple scattering. The measurements were conducted with the Zetasizer (Nano ZS, Malvern Instruments Ltd., UK). *d*_*z*_ was calculated from the autocorrelation function of the intensity of light scattered from the particles. The software used was Zetasizer Software 7.13, supplied by the manufacturer (Malvern Instruments Ltd.) Disposable poly(styrene) cuvettes (ZEN0040) were used for sample measurements. Phase-separated nanoemulsions were re-mixed by shaking prior to dilution for DLS measurements.

### Emulsion droplet zeta potential distributions

The rate of droplet movement under the influence of an external oscillating electrical field with a voltage of 150 V (electrophoretic mobility) was measured with the Zetasizer (Nano ZS, Malvern Instruments Ltd., UK) in folded capillary zeta-cells obtained from Malvern (DTS1070). The measured electrophoretic mobilities were converted to *ζ*-potentials by the instrument software (Zetasizer Software 7.13, Malvern Instruments Ltd., UK) using Henry’s equation:

1 $$ U_{e} = \frac{2\varepsilon\zeta}{3\eta}.f_{a\kappa}  $$

where *U*_*e*_ is the electrophoretic mobility, *ε* is the dielectric constant, *ζ* is the zeta potential, *η* is the viscosity of the dispersant, and *f*_*aκ*_ is the Henry function. The Smoluchowski approximation, *f*_*aκ*_ = 1.5, was used for high ionic strength media, given that water was the bulk phase in all the measured emulsions (Sze et al. 2003).

### Optical microscopy

For optical microscopy, 10 *μ*L of the nanoemulsion was placed on a clean glass slide. A glass cover-slip was then positioned upon the droplet, and sealed in place with transparent nail-polish. Samples were examined and photographed immediately under an Olympus BX-51 microscope equipped with an Olympus U-CAMD3 camera under bright-field conditions at a magnification of 50x.

### Scanning electron microscopy (SEM)

SEM was performed on an FEI Nova NanoSEM430. Nanoemulsion droplets were dispersed on polished silicon (100) chips and dried, followed by sputter coating to 5–6 nm with iridium using a Lecai EM ACE600 system. For cryo-SEM, an FEI Helios NanoLab 650 FIB/SEM system equipped with Quorum PP3010T cryo system was used. Sample imaging temperature was -140^∘^C, and sublimation was -70^∘^C for 10 min. SEM images were analyzed using ImageJ (Abramoff et al. 2004).

### Stress testing

As a metric of stress-induced nanoemulsion instability, we calculated % change in nanoemulsion droplet sizes (*d*_*z*_) as follows:

2 $$ \%\Delta d_{z} = \left(\frac{d_{z,f} - d_{z,i}}{d_{z,i}}\right) \times 100  $$

where *d*_*z*,*f*_ is the *z*-average nanoemulsion droplet size after the application of the stressor, and *d*_*z*,*i*_ is initial value for *d*_*z*_.

The same quantity for variations in *ζ* potential values is defined as follows:

3 $$ \%\Delta \zeta = \left(\frac{\zeta_{f} - \zeta_{i}}{\zeta_{i}} \right) \times 100  $$

where *ζ*_*f*_ is the *ζ* potential of the nanoemulsion after the application of the stressor, and *ζ*_*i*_ is initial value. For the purposes of this study, negative values of *%Δζ* indicate destabilization by shifting of the *ζ* potential value towards zero from either direction, while a positive *%Δζ* indicates a shift to numerically greater *ζ* potential values with attendant increase in interdroplet repulsions.

#### Long-term storage

To examine the effect of long term storage on the colloidal and chemical stability of the nanoemulsion, we stored them in tightly capped amber glass vials in a refrigerator at 4^∘^C. Aliquots were periodically removed from the vials and diluted prior to DLS measurements. Measurements were performed immediately after high pressure homogenization, and after that, once every seven days for up to six weeks.

#### Flash heating

1 g of the optimized nanoemulsion was placed in a preheated water bath and the internal temperature of the nanoemulsion was maintained at 80^∘^C for 1 minute. This protocol is a more extreme version of the high-temperature short-time (HTST) pasteurization process (typically, 71.5^∘^C for 15 s) that fruit juices and milk beverages are subjected to in the industry (Negiz et al. 1998). The nanoemulsion was then allowed to cool to room temperature, and a part of it was diluted for DLS study. The rest was retained for cannabis potency evaluation.

#### Freeze-thaw cycle

1 g of the optimized nanoemulsion was placed in a freezer at a temperature of -20^∘^C for 1 hour; then, the nanoemulsion was removed from the freezer and allowed to revert to room temperature. A part of the thawed nanoemulsion was diluted for DLS study, and the rest was retained for cannabis potency evaluation.

#### Dilution and carbonation

In a representative study, 1 mL of a representative cannabis nanoemulsion containing *ca.* 10 mg CBD was diluted to 355 mL (volume of a standard beverage can) using HPLC-grade water, thereby setting the CBD content at 10 mg per packaging unit. The diluted emulsion was then subjected to dynamic light scattering measurements. Given the absence of any stressors that might lead to cannabis decomposition or physical separation during this procedure, a potency evaluation wasn’t performed. It is to be noted that the diluted nanoemulsion was almost transparent, which is a property sought by manufacturers of cannabis-enriched carbonated-water type beverages (Donsi 2018; Saari and Chua 2020).

For the carbonation study, a similar protocol was adopted; however, for the dilution of 1 mL nanoemulsion concentrate to 355 mL, carbonated water obtained from a domestic $\text {Sodastream}^{\circledR }$ unit operating in the “medium level of carbonation” mode was used. The resultant diluted beverage, containing approximately 10 mg of CBD, had a pH of 3.8.

#### Change in pH

1 g aliquots of the optimized CD_*CBD*_ nanoemulsion were withdrawn at a starting pH of 3.6, and the pH of these aliquots were adjusted by the addition of 0.25 or 0.025(M) HCl or NaOH solution to values ranging from 1.9 to 9.4. The final weights of all aliquots were adjusted to the same value with HPLC water. After an incubation period of 12–18 h, these were diluted with solutions of matched pH values and subjected to DLS studies for measurement of average droplet diameters and zeta potentials. Meanwhile, cannabis potency evaluations were performed on the undiluted pH-adjusted aliquots.

#### Ionic and covalent additives

In a representative experiment, varying masses of CaCl_2_·2 H_2_O were added to 5 mL portions of the optimized nanoemulsion so as to span a salt concentration range between 1 and 250 mM. To ensure complete dissolution of the salt, the aliquots were vortexed for 1 min each. After an incubation period of 12–18 h, it was noted that in vials with [ CaCl_2_·2 H_2_O] ≥100 mM, the nanoemulsions had partially or totally phase-separated (Figure S3). In order to perform the DLS studies, the aliquots were vortexed again for 1 min prior to dilution with calcium chloride solutions.

A similar protocol was followed to observe the impact of the addition of model non-ionic additive (here, sucrose, a sweetener) to a CD_*CBD*_ nanoemulsion. To 5 mL portions of the optimized nanoemulsion were added varying masses of sucrose so as to span a sucrose concentration range between 1 and 500 mM (for reference, a 355 mL can of Coca-Cola typically has 39 g of sugar (The Coca-Cola Company 2021), which amounts to a sugar concentration of *ca.* 320 mM, assuming all the sugar is sucrose). To ensure complete dissolution of the sucrose, the aliquots were vortexed for 1 min each. After an incubation period of 12–18 h, 1 mL of aliquot was withdrawn from each sample in preparation for DLS evaluation. It is to be noted that cannabis potency studies were not carried out in the context of salt and sugar addition to the nanoemulsions, given that neither is expected to degrade cannabinoids and any cannabis potency loss from the bulk of the solution may be attributed to phase separation rather than to chemical transformations.

### Quantification of cannabis by HPLC

Quantitative cannabinoid analysis was performed at RPC, New Brunswick’s provincial research organization (PRO), a research and technology organization (RTO) offering contract research and technical services in Fredericton. Cannabinoids were analyzed using an Agilent 1200 series HPLC system. Separation of cannabinoids was performed with a Phenomonex C18 column (Luna 5 *μ*m, 5.6 mm I.D. X 150 mm). The mobile phase was acetonitrile (A) and 25 mM ammonium formate solution (pH 3.75) (B) at a flow rate of 1 ml/min. The isocratic flow was 80% (A), 20% (B). The diode array detector was set at 228 nm. All solvents used were HPLC-grade and filtered via 0.20- *μ*m filters.

For calibration purposes, the cannabinoid standards *(vide supra)* were mixed at concentrations of 1 ppm, 2 ppm, 10 ppm, 20 ppm and 100 ppm. For analysis, 100 mg of sample was extracted with 30 mL of 90:10 methanol:chloroform, vortexed for 20 seconds and placed in an ultrasonic bath for 20 minutes. Afterwards, sample was centrifuged at 1200 rpm for 5 minutes. Supernatant was filtered using a 0.20 *μ*m filter, and fed into the HPLC.

### Statistical analysis

All DLS measurements were carried out in triplicate on at least two samples. Mean and standard deviations were calculated using Microsoft’s Excel spreadsheet package (2016). For zeta potentials, the error bars represent the zeta deviation values obtained directly from the instrument.

## Results and discussion

### Optimization of emulsifier wt%

**Fig. 2:**
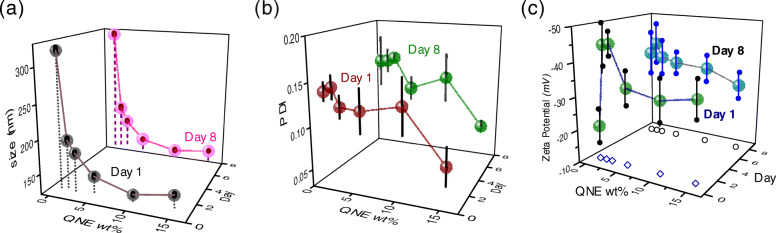
Variation of **a** *d*_*z*_, **b** PDI, and **c** *ζ* potential of a 10 wt% soybean oil nanoemulsion, immediately upon fabrication, and after a week, as a function of the wt% of $\text {QNaturale}^{\circledR }$ (QNE) in the nanoemulsion

In nanoemulsions, the use of the least possible amount of emulsifier necessary to create a stable emulsion is recommended (*vide supra*). This is one of the primary justifications for the use of $\text {QNaturale}^{\circledR }$ (15 wt% quillaja saponin) in our nanoemulsions: its low molecular mass and facile reduction of interfacial tension (*ca.* 5 mN/m for MCT oil/water interface at 1 wt% quillaja saponin) leads to its rapid absorption at the lipid/water interface, and consequent stabilization of the resultant emulsions, even at low emulsifier concentrations (Chung et al. 2017). It is, however, of utmost importance to optimize the emulsifier wt%. We synthesized 10 wt% soybean oil nanoemulsions with 0.5, 1.5, 2.5, 5, 10, and 15 wt% of $\text {QNaturale}^{\circledR }$, and after 10 homogenization cycles at 30 Kpsi, observed the lipid phase droplet sizes and *ζ* potentials (Fig. 2a). The difference between the average diameters of the lipid phase droplets of a nanoemulsion containing 0.5 wt% emulsifier and one containing 1.5 wt% emulsifier was about 125 nm. In contrast, when the emulsifier concentration was varied from 5 wt% to 10 wt%, this difference was only about 12 nm. Upon further increasing the $\text {QNaturale}^{\circledR }$ concentration to 15 wt%, the emulsion clearly enters an ‘emulsifier-rich region’, and the droplet sizes become independent of the emulsifier concentration (McClements 2015). It is to be noted that the droplet sizes recorded by us (*ca.* 120 nm) matches well with the values recorded by Leibtag et al. for their olive oil/CBD/quillaja saponin nanoemulsion produced by a sonication method (*ca.* 110 nm), indicating a lower bound for quillaja saponin stabilized CBD/carrier oil droplets in the aqueous phase of a nanoemulsion, regardless of the emulsion preparation method (high pressure homogenization versus ultrasonic cavitation) or carrier oil type (soybean oil versus olive oil) (Leibtag and Peshkovsky 2020). It was suggested that this lower bound was a function of the character and packing capabilities of the surfactants. Similarly, more negative *ζ* potential values were recorded at intermediate $\text {QNaturale}^{\circledR }$ concentrations. The samples also showed enhanced monodispersity at greater emulsifier concentrations (Fig. 2b). These quantities, upon being remeasured after a week of storage under ambient conditions, did not exhibit any major deviations from their original values, other than demonstrating a smaller dependence of the *ζ* potential on the QNE wt%. For further experiments, we selected a $\text {QNaturale}^{\circledR }$ concentration of 10 wt%, which roughly equated to a quillaja saponin concentration of approximately 1.5 wt% in the resultant nanoemulsion.

### Optimization of number of homogenization cycles

**Fig. 3:**
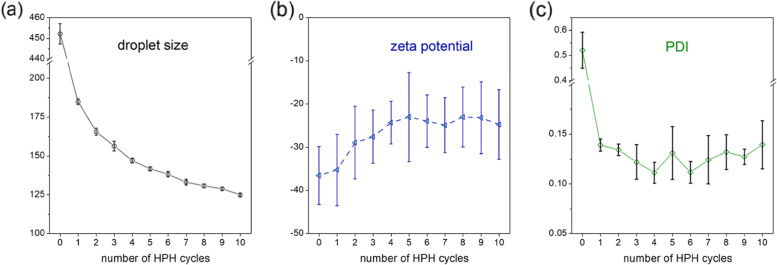
Variation of **a** *d*_*z*_, **b** *ζ* potentials, and **c** PDI as a function of the number of homogenization cycles for a cannabis-free soybean oil nanoemulsion

**Fig. 4:**
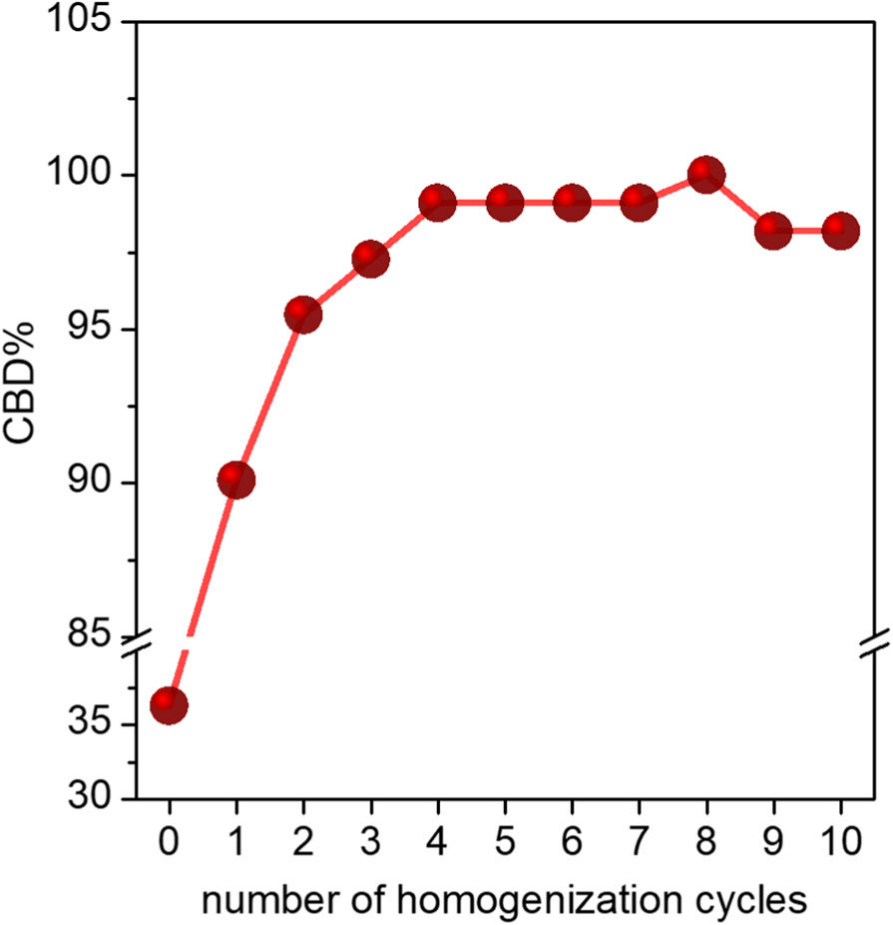
Variation of CBD% in nanoemulsion as a function of the number of homogenization cycles for a CD_*CBD*_ nanoemulsion

Before the introduction of the CD_*CBD*_ within the nanoemulsion, we attempted to optimize factors such as the number of homogenization cycles, too few of which would lead to larger droplet sizes with attendant instability, while an excessive number would not only be operationally inefficient and expensive, but might also lead to emulsifier degradation and reduced colloidal stability of the emulsions (Peng et al. 2015). Therefore, we carried out 10 consecutive homogenization cycles at 30 Kpsi on a 10 wt% soybean oil nanoemulsion containing 10 wt% $\text {QNaturale}^{\circledR }$ (Fig. 3), sampling aliquots after each processing cycle. There was a large reduction in soybean oil droplet sizes after the initial couple of homogenization cycles - *d*_*z*_ values changed from *ca.* 450 nm for the coarse emulsion, to 185 nm after one homogenization cycle, and to 165 nm after the second homogenization cycle. After this, the size largely stabilized; in fact, droplet size differences were within the limits of experimental error after 8–9 homogenization cycles. Therefore, we selected 10 as the optimal number of homogenization cycles for the generation of stable nanoemulsions. The entire high-pressure homogenization process for a coarse emulsion weighing *ca.* 40 g took 9.6 minutes for 10 homogenization cycles. *ζ* potential values of the droplets showed a slight reduction (*ca.* 10 mV) over this range, possibly owing to detachment of charged moieties from the structure of quillaja saponin, caused either by the homogenization itself, or by the resultant localized heating within the emulsion during the process (Rampon et al. 2003; Floury et al. 2003). This experiment was repeated with the optimized cannabis nanoemulsion (*vide infra*) in order to evaluate the impact of the number of HPH cycles on cannabis potency (Fig. 4), with the measured weight of the CBD used in the emulsion preparation taken as 100%. Considering the CBD concentration in the nanoemulsion as a metric of cannabis potency, we found that the CBD concentration did not change beyond the limits of experimental error after the first two cycles, thereby indicating that CBD decomposition over 10 homogenization cycles is not a concern (Fig. 4). Incomplete mixing of the two phases and/or slight phase separation upon storage leading to heterogeneity in the samples explained the lower [CBD] values in the samples corresponding to the first two homogenization cycles analyzed by HPLC. Variation of the optimized nanoemulsion droplet sizes with the number of homogenization cycles were comparable to those recorded for the cannabis-free nanoemulsion (Figure S2).

### Optimization of lipid phase composition

**Fig. 5:**
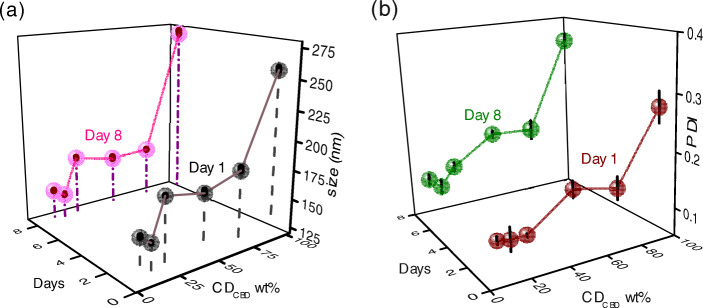
Variation of **a** *d*_*z*_ and **b** PDI of a phytocannabinoid nanoemulsion, immediately upon fabrication, and after a week, as a function of the wt% of CD_*CBD*_ in the lipid phase of the nanoemulsion

In preliminary experiments, we noticed that cannabis-heavy lipid phases were more viscous and in general more difficult to handle. Moreover, nanoemulsions made with cannabis-majority lipid phases had broad, bimodal droplet size distributions and tended to exhibit droplet agglomeration upon prolonged storage. Therefore, we fabricated a series of lipid phases with varying CD_*CBD*_ contents (100, 75, 50, 25, 16.6, and 10 wt% of CD_*CBD*_) mixed with appropriate amounts of soy oil. These were then used to generate 10 wt% lipid phase nanoemulsions containing 10 wt% $\text {QNaturale}^{\circledR }$ as described before. Dispersed phase droplet sizes, polydispersity index values, and *ζ* potentials were measured for these systems both immediately after creation, as well as after 7 days under ambient storage conditions. It is evident that the average lipid phase droplet sizes decrease (Fig. 5a) and the dispersion becomes monodisperse (Fig. 5b, Figure S2) as the cannabis oil wt% is reduced, potentially owing to the higher viscosity of the cannabis extract compared to soybean oil. The *ζ* potentials, on the other hand, remain relatively unchanged within the limits of experimental error, both as a function of the lipid phase composition, as well as upon prolonged storage (Table 1). Further experiments were conducted with the lipid phase containing 16.6 wt% of CD_*CBD*_.

**Table 1:** *ζ* potential values of CD_*CBD*_ nanoemulsions with varying lipid phase compositions

Day	CD_*CBD*_% ^a^	*ζ*(mV)	*ζ* deviation (mV)
1	10	-33.6	7.6
1	75	-28.1	5.7
8	10	-30.6	7.1
8	75	-21.1	7.4

^a^Percentage CD_*CBD*_ in the lipid phase; soybean oil is the other lipid phase component

### Imaging the optimized cannabis/soybean oil-in-water nanoemulsion

**Fig. 6:**
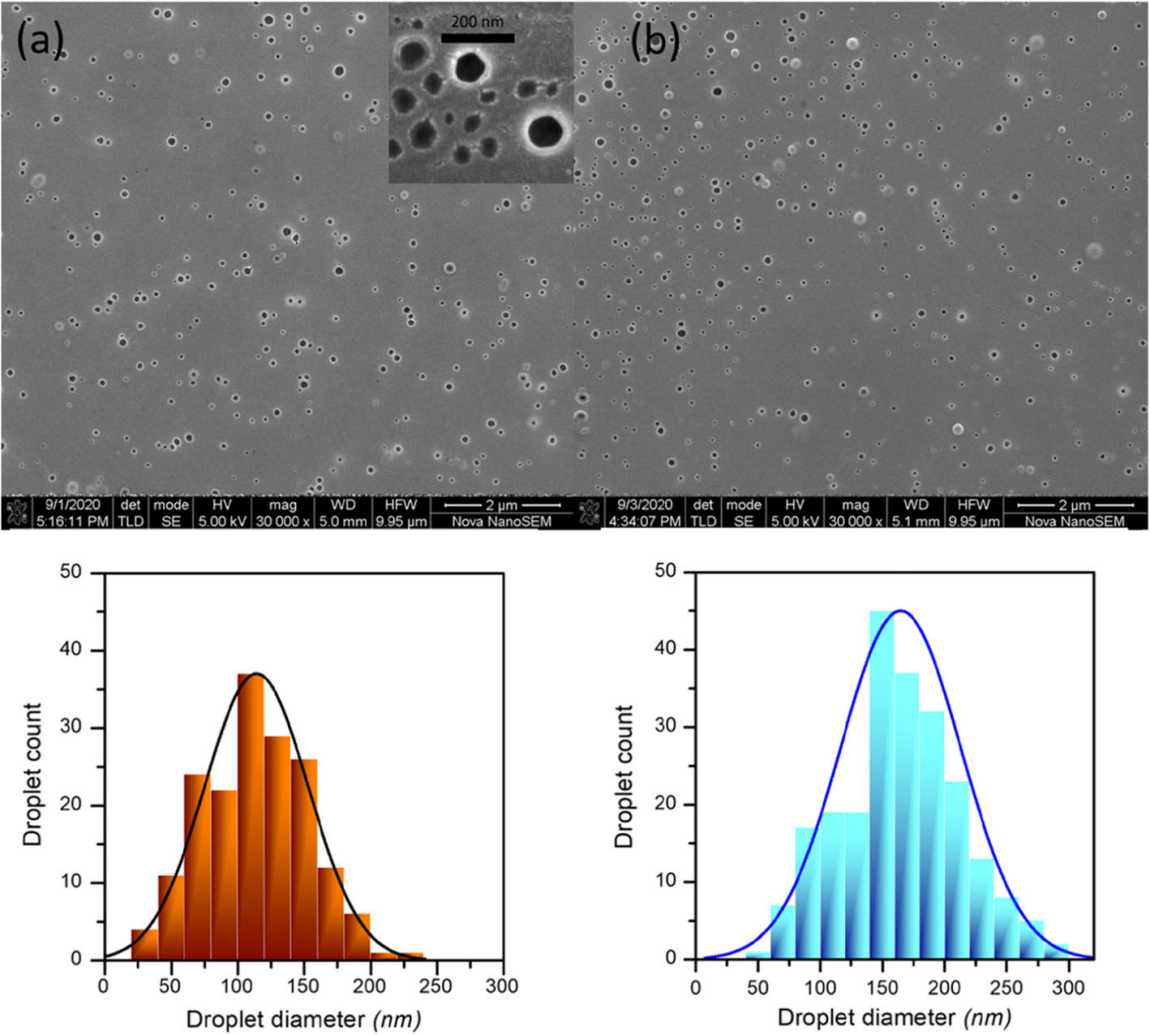
**a** SEM of the optimized nanoemulsion, showing lipid phase droplets; (inset) close-up of the nanoemulsion droplets; **b** SEM of the nanoemulsion in the presence of 50 mM CaCl_2_. Size distribution profiles are underneath the respective SEMs

Representative SEMs of the optimized CD_*CBD*_ nanoemulsion may be found in Fig. 6. The size distribution, calculated from data obtained by measuring the diameters of >250 droplets, revealed the average droplet size to be 115 nm (SD= 39 nm), which is somewhat smaller than the DLS size data. This, however, is not unexpected, since DLS tends to ‘over-weigh’ larger particles owing to their greater light-scattering ability (Souza et al. 2016). Moreover, DLS considers a layer of water molecules surrounding the lipid droplet, generating what is called the hydrodynamic radius, which is inevitably greater than the actual radius of the lipid droplet.

In the presence of 50 mM CaCl_2_·2 H_2_O, the nanoemulsion droplets show an increase in size, with the average droplet diameter increasing to 165 nm (SD= 48 nm); this trend is in agreement with the DLS data. Optical micrographs of the optimized nanoemulsion with and without additives maybe found in Figure S5 as well as insets in the appropriate graphs.

### Impact of physical stressors

**Fig. 7:**
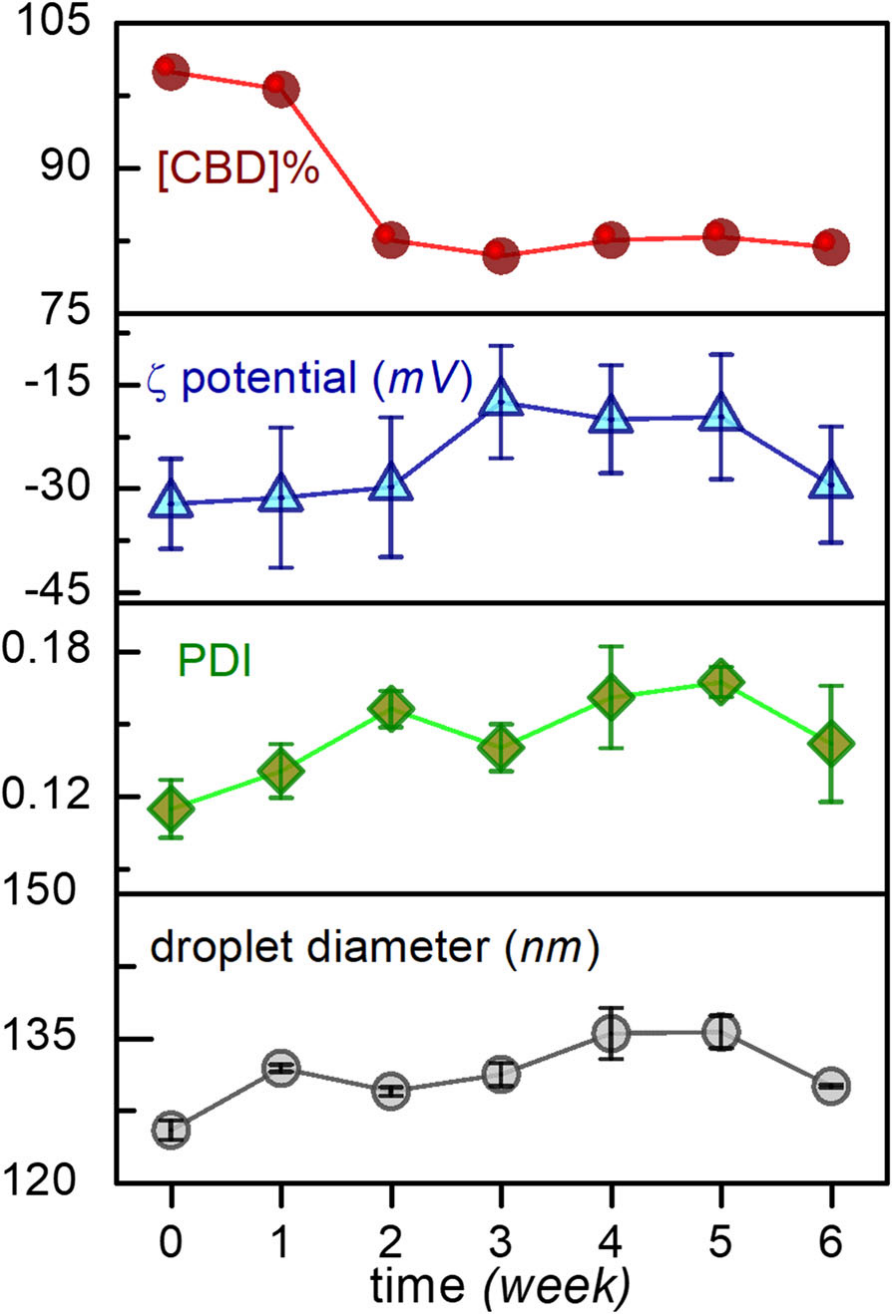
Variation of CBD concentration (as a % of measured CBD used in nanoemulsion preparation) in the nanoemulsion, zeta potentials, PDI, and droplet sizes as a function of the number of weeks of nanoemulsion storage

The impact of physical stressors on the stability and potency of cannabis in the optimized cannabis nanoemulsion have been summarized in Table 2, with the *d*_*z*,*f*_ and *ζ*_*f*_ values obtained from measurement of control nanoemulsion samples *sans* exposure to stressors. On the basis of the % *Δd*_*z*_ values, we conclude that pasteurization-style flash heating and a single freeze-thaw cycle at -20^∘^C has minimal impact on the nanoemulsion parameters except a slight increase in droplet sizes. Dilution to 355 mL, using either still or carbonated water, reduces average droplet size slightly, while droplet size distribution profiles remain unaltered, which confirms that our nanoemulsion is kinetically metastable. However, upon storage of the nanoemulsion for 6 weeks under ambient conditions, slight ’ringing’ (creation of a very thin whitish creamy ring on the glass surface at the top of the emulsion) (Kumar and Sarkar 2018) was observed, likely due to difference in density between the oil and water phases, and the *ζ* potential values fell. Concentration of CBD in the optimized nanoemulsion decreased by *ca.* 17% after 6 weeks of storage upon comparison to the initial concentration of CBD in the freshly synthesized nanoemulsion (Fig. 7).

**Table 2:** Impact of stressors on optimized CD_*CBD*_ nanoemulsion parameters

System	Stressor	*d*_*z*,*f*_ (nm)	*%Δd*_*z*_	*ζ*_*f*_ (mV)	*%Δζ*_*f*_	[CBD] (mg.g ^−1^)
1^a^	none	125.5	N/A	-32.2	N/A	10.8
2	heat	136.8	9	-30.9	-4	10.4
3	freeze-thaw	142.5	13.5	-30.2	-6.2	10.6
4	dilution^b^	123.6	-1.5	-25.6	-19.7	N/A^c^
5	dilution/carbonation^d^	135.8	-3.5	-25.0	-8.9	N/A^c^
6	6 weeks storage	130.0	3.6	-29.4	-8.7	9.1

^a^Optimized nanoemulsion, as synthesized

^b^1 mL nanoemulsion added to HPLC water to obtain a final volume of 355 mL

^c^[CBD] not measured as dilution is not expected to change it

^d^1 mL nanoemulsion added to carbonated water to obtain a final volume of 355 mL

### Impact of chemical stressors

In practical applications, cannabinoid nanoemulsions are expected to retain their desirable emulsion properties over a wide range of pH values and ionic strengths, depending upon product composition. Factors such as pH, the addition of ionic salts, and organic additives such as sugars can have profound effects on the stability of nanoemulsions (McClements 2004).

#### Effect of pH

**Fig. 8:**
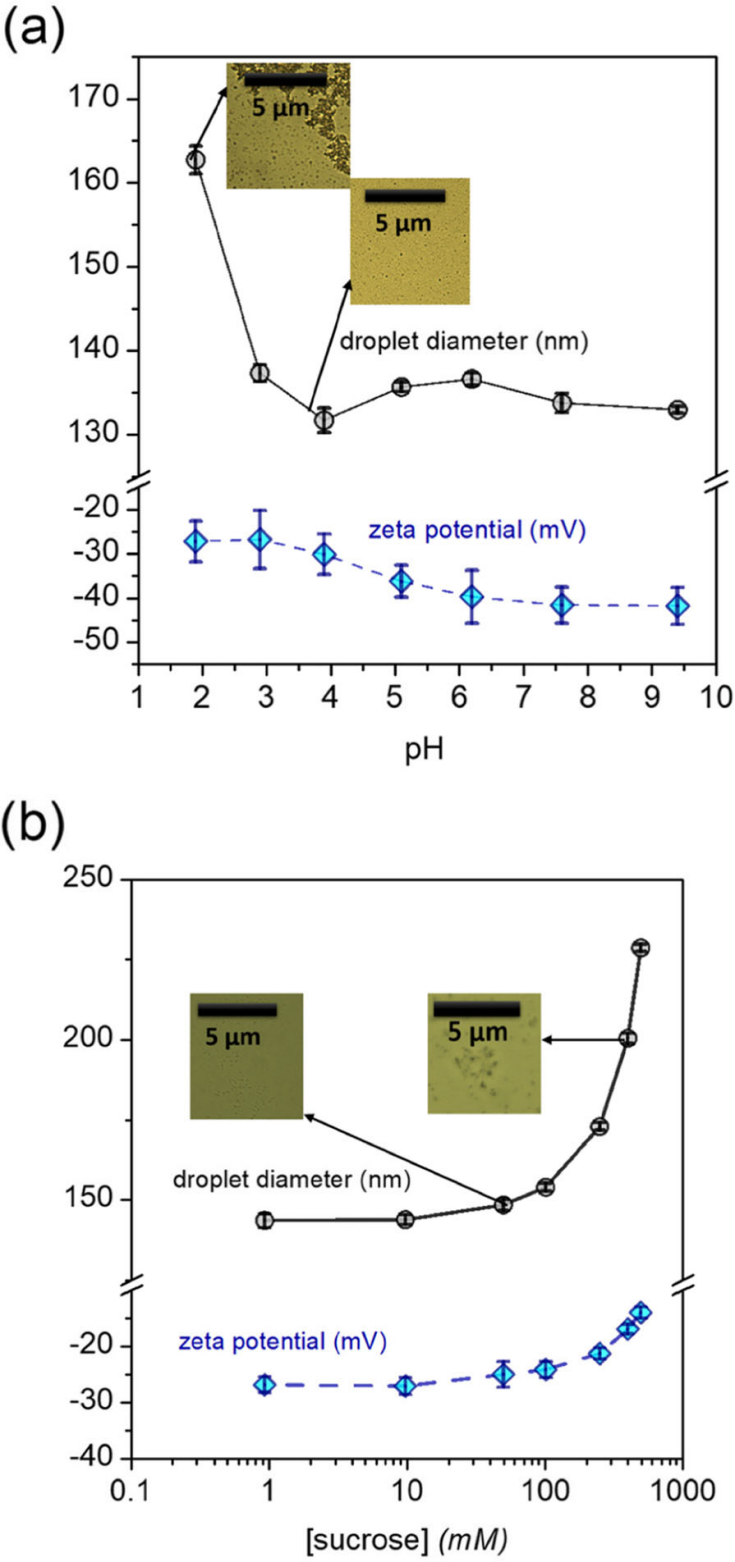
Variation of *d*_*z*_ and *ζ* potential of the optimized CD_*CBD*_ nanoemulsion as a function of: **a** the pH; **b** increasing sucrose concentration. (Inset) optical micrographs of the optimized nanoemulsion **a** at pH 3.8 and pH 1.6; **b** at two different sucrose concentrations

Changes in pH can protonate or deprotonate terminal functional groups of emulsifiers, thereby modulating interdroplet electrostatic repulsive forces, and ultimately affecting droplet coalescence rates (Chung et al. 2017). For our optimized nanoemulsion, however, a moderate increase in droplet size (*ca.* 30 nm) was observed upon lowering the pH to highly acidic regime (pH < 2), while no changes in droplet sizes were recorded upon raising the pH to 9 (Fig. 8a). Visual observation of the emulsions indicated that creaming occurred at pH 2 and below, which is consistent with previous reports (Yang et al. 2013); also, optical microscopy of the optimized nanoemulsion at pH 1.6 revealed the presence of extensive flocs. These results seem to indicate that despite minimal particle coalescence, the emulsions show a high degree of flocculation at pH 2 and below. Protonation of the terminal -COO ^−^ groups of quillaja saponin leading to reduced interdroplet repulsion at very low pH values may be invoked to explain this observation. As expected, zeta potentials became steadily more negative with increase in pH. These results indicate that CD_*CBD*_ nanoemulsions stabilized by quillaja saponin are stable across most of the pH range present within food products (pH 3–8), but very acidic matrices might lead to flocculation in such nanoemulsions. Quantitative analysis of emulsions at various pH values indicated a steady concentration of CBD that didn’t change at different pH values, even after a month of incubation in the fridge at 4^∘^C, suggesting that flocculation in this case is not accompanied by irreversible phase separation (Figure S6).

#### Effect of covalent additives

The addition of varying amounts of sucrose, a commonly used sweetener, initially caused no changes to the CD_*CBD*_ nanoemulsion droplet sizes and *ζ* potential values; however, lipid droplet sizes begin to increase when the sucrose concentration exceeds 250 mM (Fig. 8b). At 500 mM sucrose concentrations, the average droplet sizes of the optimized CD_*CBD*_ nanoemulsion increased from *ca.* 130 nm to *ca.* 170 nm. From cryo-SEM, average lipid phase droplet size of the optimized nanoemulsion in the presence of *ca.* 100 mM sucrose was found to be 182.3 ±48.4 nm, which is in rough agreement with DLS size data (Figure S4). This observation is explained by the growing disparity between the density and viscosity of the aqueous and lipid phases as more and more sucrose is dissolved in the aqueous phase (McClements 2015). The *ζ* potential values of the nanoemulsion showed a slight increase (*ca.* 10 mV) with increase in sucrose concentration; this was seen to be reproducible over multiple experiments. This was likely brought about by the expanding location of the slipping plane during electrophoretic movement with increasing concentration of sugars, due to the formation of a viscous hydration layer on the surface of the nanodroplets, as recorded previously by Matsumoto (1994). No noticeable changes in the polydispersity index of the samples were noticed even at the highest sucrose concentrations.

#### Effect of ionic additives

**Fig. 9:**
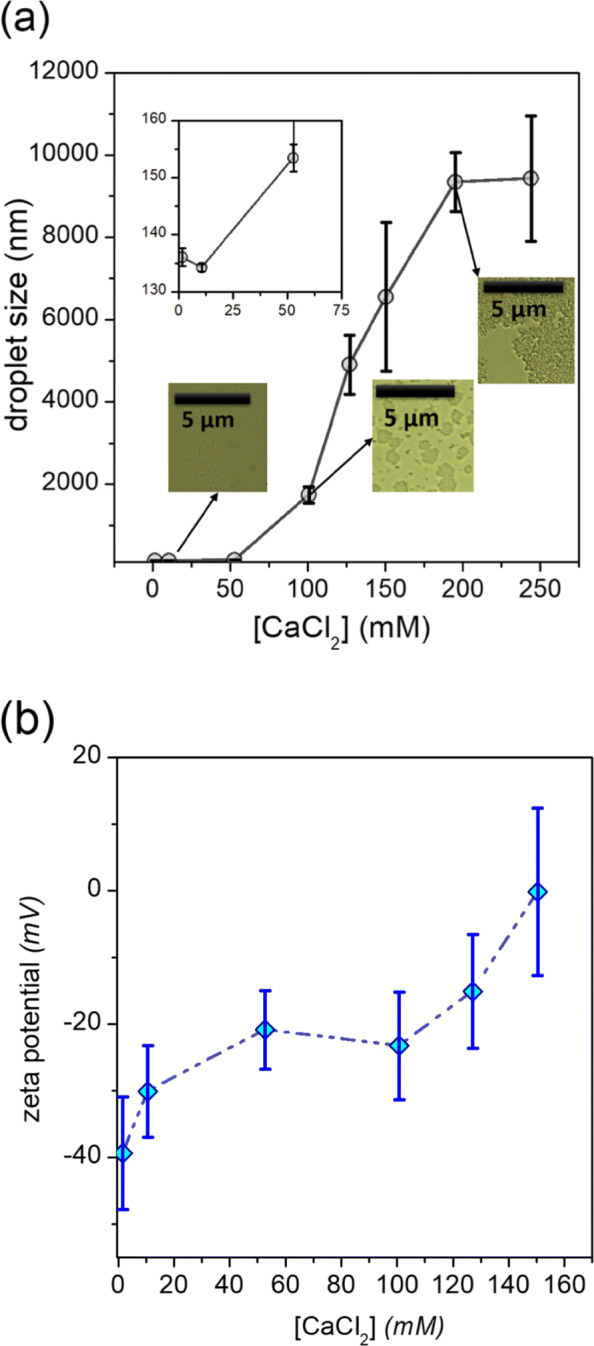
**a** Variation of *d*_*z*_ of the optimized CD_*CBD*_ nanoemulsion as a function of increasing calcium chloride concentration; (inset) optical micrographs of the optimized nanoemulsion in the presence of varying concentrations of CaCl_2_ ; **b** Variation of *ζ* potential of the optimized CD_*CBD*_ nanoemulsion as a function of increasing calcium chloride concentration

The addition of CaCl_2_·2 H_2_O was intended to mimic the effect of hard water; also, essential minerals are often intentionally added to nutraceutical products (Keowmaneechai and McClements 2002). It is already known that the addition of salts such as CaCl_2_·2 H_2_O can lead to instability and phase separation in nanoemulsions containing quillaja saponin; nevertheless, it is superior in this context to other emulsifiers such as beet pectins, given that it offers both electrostatic and steric stability to the lipid phase droplets (Ralla et al. 2017). Previous studies have demonstrated the quillaja saponin stabilized nanoemulsions showed droplet aggregation and flocculation at [NaCl] ≥300 mM (Ralla et al. 2017). In our study, we found that CD_*CBD*_ nanoemulsion droplet sizes remained in the sub- *μ*m region despite some increase in average diameters at [ CaCl_2_·2 H_2_O] ≤50 mM (Fig. 6b); at 100 mM, slow but discernible phase separation was initiated, while above 100 mM rapid separation of nanoemulsion into serum layer and creamy layer could be observed (Figure S3). The primary reason for this observation is the high screening capacity of the Ca ^2+^ ion, which reduces the interdroplet Debye screening length, and consequently interdroplet repulsion, until attractive interactions between droplets (such as van der Waal forces) predominate, leading to droplet coalescence and/or flocculation (McClements 2015). This effect is supposed to be especially strong in emulsions with *ζ*-potentials with magnitudes above 25 mV (as is the case here) in the presence of polyvalent cations (McClements 2015; Ralla et al. 2017) (Fig. 9a). We attempted to measure the *ζ*-potentials of our CaCl_2_-containing nanoemulsion aliquots,(Fig. 9b) but the high ionic strengths led to large errors in measured *ζ*-potential values, possibly owing to effects such as ion-droplet and droplet-ion-droplet bridging (Dickinson 2010). It is clear, however, that drastic reduction of *ζ*-potential could be observed with increasing [ CaCl_2_·2 H_2_O], with values dropping to zero between 150 and 200 mM [ CaCl_2_·2 H_2_O] (Fig. 9b). In order to visualize our optimized nanoemulsion in the presence of various additives, optical microscopy was carried out, and the respective micrographs may be found in the SI (Figure S5).

The relatively high stability of the optimized CD_*CBD*_ nanomeulsion may be attributed to the fact that the creaming rate is directly proportional to the square of the droplet diameter - an emulsion with nanosized droplets, therefore, has a reduced rate of gravitational separation (McClements 2015). Additionally, Brownian motion effects that favor a homogeneous distribution of droplets throughout the system counteract the gravitational forces when the droplets are sufficiently small, as is the case for our optimized nanoemulsion. The charged -COO ^−^ termini present in the quillaja saponin structure also offers electrostatic stabilization by enhancing inter-droplet repulsion. With respect to their chemical stability, the documented high free radical quenching capacity of quillaja saponin may help minimize oxidative degradation of CD_*CBD*_ within the emulsion lipid phase droplets (Uluata et al. 2016; Tippel et al. 2017).

## Conclusions

This report presents the design, creation, and stress-testing results for a novel cannabidiol-enriched nanoemulsion as a representative cannabis-containing nutraceutical suitable for ingestion. After optimization of the number of high pressure homogenization cycles, emulsifier wt%, and lipid phase composition, we produced a CBD-enriched soybean oil nanoemulsion in water in the presence of 1.5 wt% quillaja saponin with average lipid phase droplet sizes of *ca.* 120 nm and *ζ* potential values of approx. -30 mV. The nanoemulsion proved to be stable over a period of 6 weeks, with minor creaming, and was resistant to potential instability owing to flash-heating, cooling, dilution and addition of carbonated water. Droplet agglomeration became a concern, however, at pH < 2, as well in the presence of excessive amounts of additives ([sucrose] ≥250 mM, [CaCl_2_] > 100 mM). Cannabis potency, measured in terms of CBD concentration, changed by ∼17% over a period of 6 weeks, and was largely unaffected by other examined stressors. It is expected that this report will be the first of a series stemming from a detailed study of various cannabinoid nanoemulsions suitable for the nutraceutical industry in Canada and other countries where the therapeutic potentials of cannabis are finally being widely acknowledged and commercialized.

## Supplementary Information

**Additional file 1** See additional file named **SI.pdf** for quantitative analysis of the CBD-enriched cannabis distillate used in this study, droplet size distribution profiles for CD_*CBD*_ nanoemulsions at various lipid phase compositions, variation of the droplet sizes of the optimized nanoemulsion as a function of the number of homogenization cycles, photographic representation of nanoemulsion destabilization and phase separation in the presence of added CaCl_2_, cryo-SEM of the optimized nanoemulsion in the presence of 100 mM sucrose, and data for the variation of CD_*CBD*_ concentration in the optimized nanoemulsion as a function of pH.

## Data Availability

The authors maintain all raw data for a minimum of 15 years and this is available from the senior corresponding author upon reasonable request. Additional data is provided as supplementary information in the accompanying files.

## Abbreviations

CBD: Cannabidiol, IUPAC name: 2-[(1R,6R)-3-methyl-6-prop-1-en-2-ylcyclohex-2-en-1-yl]-5-pentylbenzene-1,3-diol
CD_*CBD*_: CBD-enriched cannabis distillate
CaCl_2_·2 H_2_O: Calcium chloride dihydrate
DLS: Dynamic light scattering
HPLC: High-performance liquid chromatography
PDI: Polydispersity index
SEM: Scanning electron microscopy
THC: *Δ*^9^-tetrahydrocannabinol, IUPAC Name: (6aR,10aR)-6,6,9-trimethyl-3-pentyl-6a,7,8,10a-tetrahydrobenzo [c]chromen-1-ol
