# Supplementary material for: Synthesis, characterization and stress-testing of a robust quillaja saponin stabilized oil-in-water phytocannabinoid nanoemulsion
Source: J Cannabis Res. 2021 Sep 23;3:43. doi: 10.1186/s42238-021-00094-w (PMC8461879; doi:10.1186/s42238-021-00094-w)
Supplement: Supplementary file 1 — Additional file 1 See additional file named SI.pdf for quantitative analysis of the CBD-enriched cannabis distillate used in this study, droplet size distribution profiles for CDCBD nanoemulsions at various lipid phase compositions, variation of the droplet sizes of the optimized nanoemulsion as a function of the number of homogenization cycles, photographic representation of nanoemulsion destabilization and phase separation in the presence of added CaCl2, cryo-SEM of the optimized nanoemulsion in the presence of 100 mM sucrose, and data for the variation of CDCBD concentration in the optimized nanoemulsion as a function of pH. [file 42238_2021_94_MOESM1_ESM.pdf]

Supplementary information for
Synthesis and characterization of a robust quillaja saponin stabilized oil-in-water phytocannabinoid nanoemulsion

A. Banerjee, R. Salama

Department of Chemistry and Biochemistry, University of Windsor, 401 Sunset Ave. Windsor, ON N9B 3P4 Canada

J. Binder

Peak Processing Solutions, Tecumseh, Ontario, N0R 1L0 Canada

J. F. Trant*

Department of Chemistry and Biochemistry, University of Windsor, 401 Sunset Ave. Windsor, ON N9B 3P4 Canada

* E-mail: john.trant@uwindsor.ca

Table S1: Cannabinoid composition of CD_{CBD}

**Phytocannabinoid**	**Concentration** (mg/g)
Δ^9-Tetrahydrocannabinol	69.0
Cannabidiol	632
Cannabigerol	7.0
Cannabinol	5.3
Cannabichromene	22.3

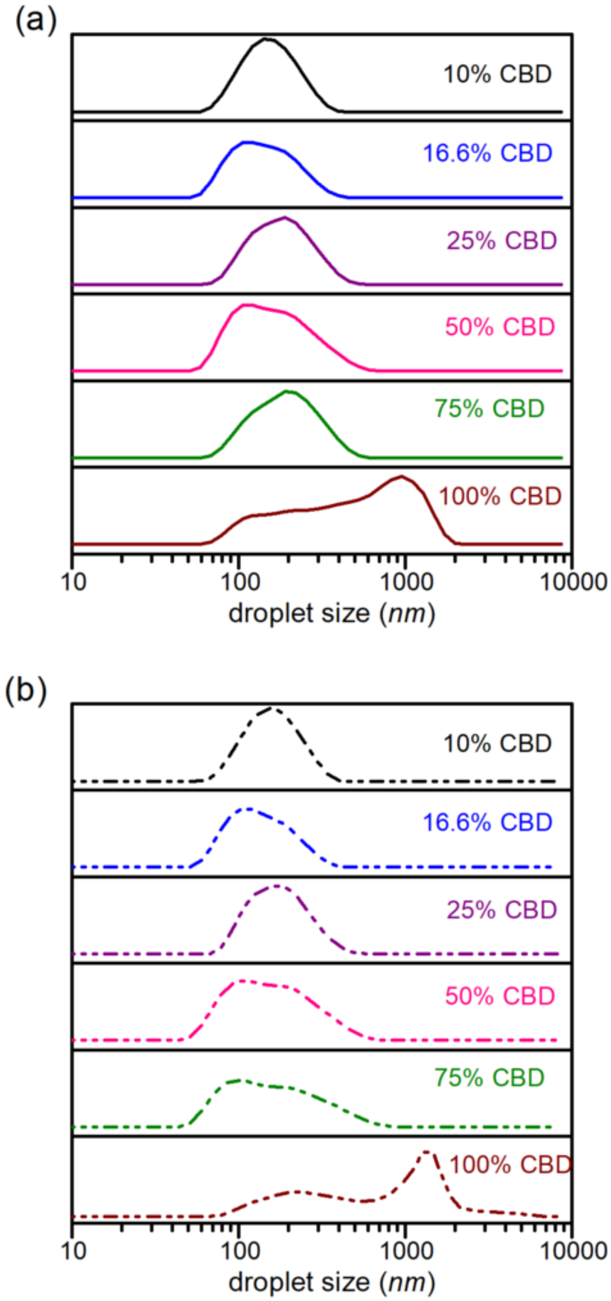

Figure S1: Droplet size distribution profile of a mixture of CD_{CBD} and soybean oil dispersed in water - **(a)** as-produced and **(b)** after one week of storage. Total lipid phase was 10 wt%, and 10 wt% QNaturale® was used as emulsifier.

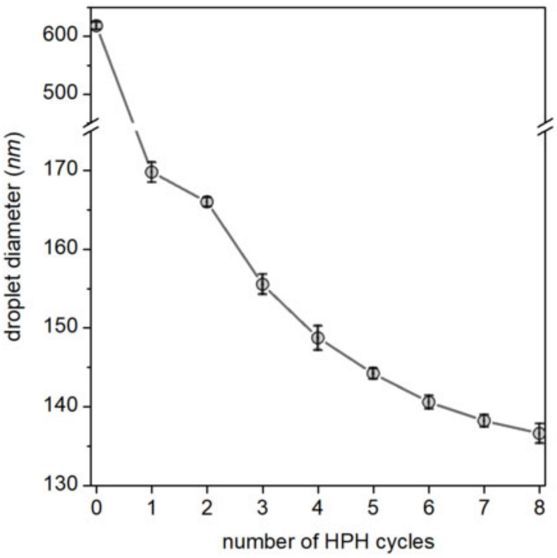

Figure S2: Variation of d_z of the optimized CD_{CBD} nanoemulsion with the number of homogenization cycles.

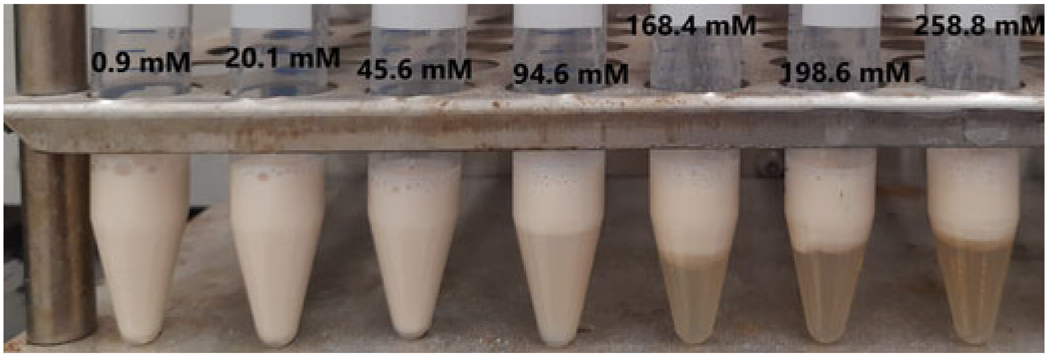

Figure S3: Optimized nanoemulsion with varying concentrations of $CaCl_2.2H_2O$ from ∼1 mM to ∼250 mM; after 18 h incubation. Exact concentrations are mentioned in the figure.

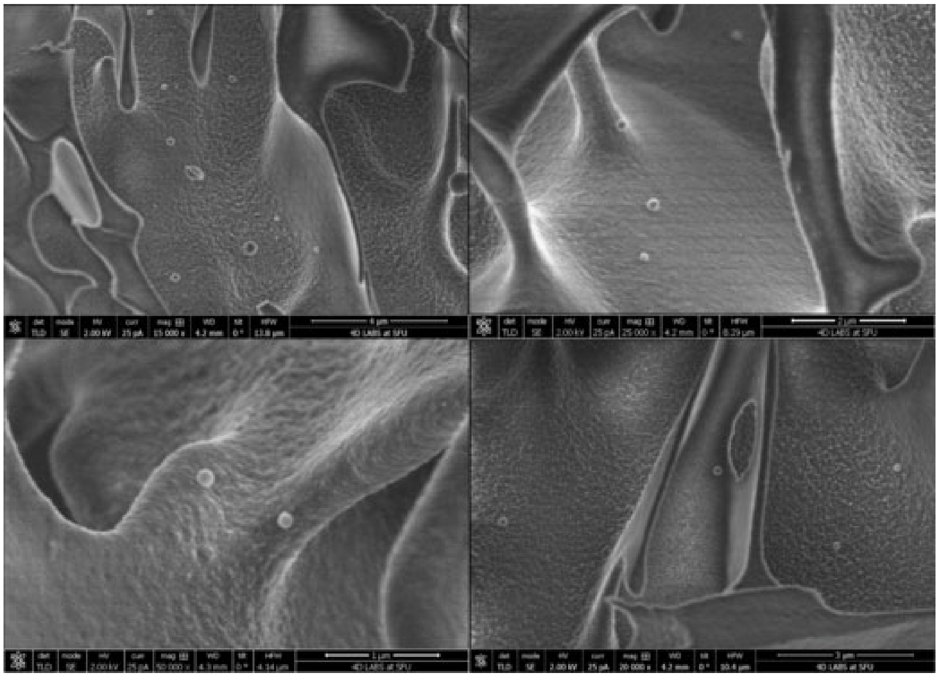

Figure S4: Optimized nanoemulsion with varying concentrations of $CaCl_2.2H_2O$ from ∼1 mM to ∼250 mM; after 18 h incubation. Exact concentrations are mentioned in the figure.

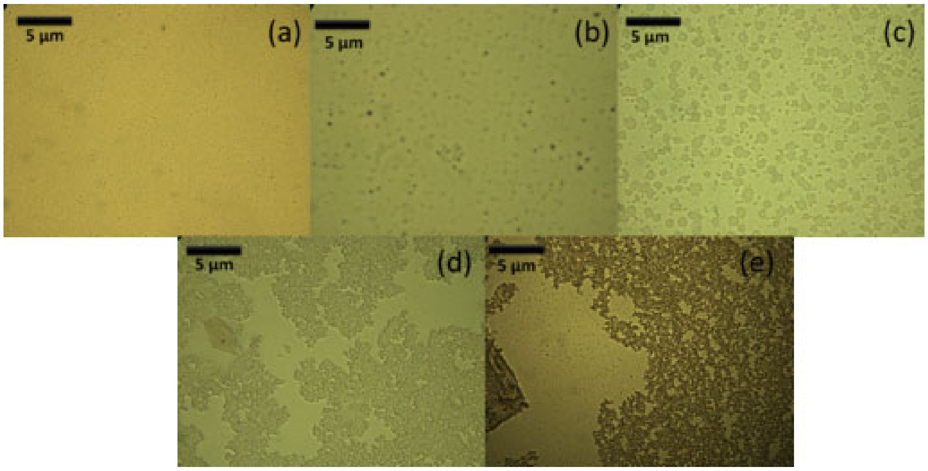

Figure S5: Optical micrographs of optimized nanoemulsion **(a)** as-prepared, at pH 3.8, without additives; **(b)** in the presence of 400 mM sucrose; **(c)** in the presence of 100 mM $CaCl_2.2H_2O$; **(d)** in the presence of 200 mM $CaCl_2.2H_2O$; **(e)** at pH 1.6.

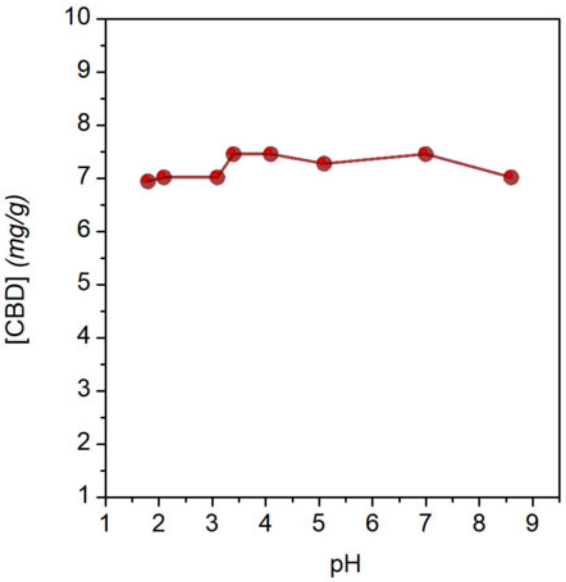

Figure S6: Variation in CBD concentration in optimized nanoemulsion as a function of pH.
